# Heat Treatment Effects on Laser Powder Bed Fused CuNi2.5SiCr Alloy: Microstructure, Hardness, Electrical, and Thermal Conductivity

**DOI:** 10.3390/ma19050883

**Published:** 2026-02-27

**Authors:** Tsovinar Ghaltaghchyan, Ani Khachikyan, Vahan Nikoghosyan, Arevik Asatryan, Marina Aghayan

**Affiliations:** 1 A.B. Nalbandyan Institute of Chemical Physics, National Academy of Sciences of the Republic of Armenia, Paruyr Sevak 5/2, Yerevan 0014, Armenia; anikhachikyan000@gmail.com (A.K.); arevik.asatryan@ichph.sci.am (A.A.); marina.aghayan@ichph.sci.am (M.A.); 2 Institute for Physical Research, National Academy of Sciences of the Republic of Armenia, Ashtarak 0204, Armenia; varan1954@gmail.com; 3 FACT Industries OU, Oisamae Tee 124-12, 13513 Tallinn, Estonia

**Keywords:** additive manufacturing, laser powder bed fusion (LPBF), CuNi2.5SiCr alloy, low laser power

## Abstract

Additive manufacturing of copper alloys remains challenging due to their high reflectivity and thermal conductivity, which limit laser energy absorption and increase energy consumption. In this study, the laser powder bed fusion of a copper-based CuNi2.5SiCr alloy is demonstrated using low laser powers (67.2–100.8 W). Samples fabricated at 67.2 W achieved a relative density of 96.8%, with no significant improvement observed at higher laser powers. The post-process heat treatments were conducted at 750 °C, 850 °C, and 950 °C to investigate their influence on microstructure, hardness, electrical and thermal conductivity. Heat treatment at 850 °C resulted in the highest hardness, improved microstructural uniformity and 20 W/mK thermal conductivity, while the as-fabricated samples exhibited the highest electrical conductivity (1.07 × 10^6^ S/m).

## 1. Introduction

The global thermal management systems market is expected to grow from $59.73 billion in 2024 to $95.64 billion by 2032, at a compound annual growth rate (CAGR) of 6.1% [1]. Copper plays a key role in this market due to its outstanding electrical and thermal conductivity, high strength, and ductility. In addition, copper offers excellent machinability, formability, and corrosion resistance [2]. Demand for complex copper components is rising, driven by the need for efficient heat dissipation, compact and lightweight designs, and reduced maintenance requirements [3]. Advances in additive manufacturing (AM) have expanded production capabilities, allowing the fabrication of intricate copper components with complex geometries [4].

Among the various AM techniques, laser powder bed fusion (LPBF) offers several advantages, including design flexibility [5], high material efficiency [6,7], rapid prototyping [8], favorable mechanical properties, and cost-effectiveness [9,10]. However, processing copper and its alloys remains challenging due to their high reflectivity and thermal conductivity, pose significant challenges for laser powder bed fusion processing, often necessitating high laser powers and specific scanning strategies to achieve adequate densification and favorable microstructures [11,12,13]. In the 1000–1100 nm laser wavelength range, pure copper can reflect up to 98% of incident energy [14], which may damage the printer optics and increase maintenance costs [15,16]. Inefficient energy absorption also reduces process efficiency, raising both economic and environmental costs. This issue can be mitigated by using shorter-wavelength green lasers to enhance copper’s optical absorption [17,18,19,20].

A novel green fiber laser operating at high pulse repetition rates and low peak power was demonstrated by Singh et al. [21] to be well suited for melting-dominated light-matter interactions. Compared to near-infrared laser sources, the green laser exhibited significantly enhanced process efficiency, enabling the fabrication of copper specimens with up to 99.8% relative densities [22].

Recent studies have explored the low and medium-energy LPBF strategies to enhance copper absorptivity while minimizing processing energy consumption [23,24]. Reducing laser power and volumetric energy density has been demonstrated to stabilize the melt pool, thereby enhancing microstructural homogeneity and dimensional precision to promote melt pool stability, thereby improving microstructural uniformity and dimensional accuracy. Nevertheless, reported studies focus on pure copper or commercial copper alloys fabricated at comparatively elevated energy densities. Systematic explorations of the low-power LPBF processing windows for precipitation-hardenable Cu-Ni-Si alloys remain limited. Moreover, the precipitation kinetics, phase transformations, and strengthening mechanisms in additively manufactured Cu-Ni-Si alloys are not fully elucidated, especially under low-energy LPBF conditions and subsequent heat treatments.

Numerous studies have investigated the application of LPBF in processing pure copper and copper alloys. Table 1 summarizes the principal research efforts in this domain.

Relative densities in the range of 88–99.6% were achieved when the pure copper was used, which typically required high laser powers (≥600 W). The high reflectivity and thermal conductivity of copper led to incomplete densification at lower powers. In contrast, Cu-based alloys consistently exhibited higher relative densities (>99%) at moderate laser powers (370–450 W) (Table 1). The addition of alloying elements enhances laser absorptivity and stabilizes the melt pool, thereby mitigating issues inherent to pure copper. This comparison underscores the importance of alloy design in improving the printability of copper materials.

The CuNi2.5SiCr alloy is a low-cost and precipitation-hardenable alloy that offers an outstanding combination of mechanical strength, thermal stability, and electrical conductivity [36,37,38]. These attributes make it a strong candidate for applications requiring both structural integrity and functional performance, such as components in aerospace, electronics, and tooling. Furthermore, the alloy undergoes precipitation strengthening through the formation of Ni_2_Si phases during the post-processing heat treatments [39]. Given these advantages, CuNi2.5SiCr represents a well-suited material choice for additive manufacturing via the LPBF technique.

Unlike prior studies requiring moderate to high laser powers (270–800 W) to produce dense copper-based alloys, this investigation demonstrates the viability of fabricating CuNi2.5SiCr using the low laser powers (<100 W). This study further establishes a systematic relationship between the low-energy LPBF processing, post heat-treatment microstructural evolution, and hardness, electrical conductivity, and thermal conductivity. This approach significantly reduces energy consumption and processing costs, representing a step toward sustainable additive manufacturing of copper alloys. Furthermore, while literature reports indicate that pure copper can achieve relative densities of ~99.9% using 1.8 kW power laser source, the corresponding microhardness is low (≈77 HV on average) [40] and 53 HV in case of relative density of up to 99.5% with a laser power of 192 W [41]. In contrast, this study illustrates that CuNi2.5SiCr components exhibiting favorable combinations of microhardness and thermal stability can be produced via laser powder bed fusion using substantially lower laser energy densities. This approach underscores the viability of fabricating high-performance, competitive parts with minimized resource utilization.

## 2. Materials and Methods

### 2.1. Powder Feedstock

This study utilized a commercially available CuNi2.5SiCr alloy powder (KME Special Products & Solutions GmbH, Osnabrück, Germany). The nominal chemical composition of the powder is listed in Table 2. The particle size distribution of the powder was characterized by D_10_ = 20 μm, D_50_ = 35 μm, and D_90_ = 60 μm. The powder demonstrated a flowability of 15 s/50 g, a bulk density of 4.1 g/cm^3^, and a moisture content below 0.01%. All the data reported above and in Table 2 were obtained from the supplier’s specifications.

### 2.2. Laser Powder Bed Fusion (LPBF)

Rectangular specimens with dimensions of 5 mm × 10 mm × 5 mm were fabricated using a Realizer GmbH SLM-50 system, Borchen, Germany, equipped with a 120 W fiber laser (Nd:YAG, wavelength 1060 nm). The LPBF process was conducted at laser powers ranging from 67 W to 101 W.

Among all the tested powers, 67.2 W produced the most uniform microstructure with comparable density while minimizing energy input; therefore, this condition was selected for subsequent heat-treatment studies. For each processing condition, five specimens were fabricated and analyzed to ensure reproducibility.

The processing parameters included a layer thickness of 40 μm, a hatch spacing of 80 μm, an exposure time of 10 μs, and a point distance of 10 μm. A pulsed scanning mode with a 60° rotation between successive layers was employed. The build plate was preheated to 100 °C, and the process was carried out under a high-purity argon atmosphere with an oxygen concentration below 0.1%.

Preheating of the build plate was employed to mitigate thermal gradients and residual stresses, thereby enhancing melt pool stability, cooling rates, and densification during LPBF processing. This approach is commonly recommended for copper and alloys LPBF and has been widely reported to improve process stability and reduce defect formation [42,43].

### 2.3. Heat Treatment

Despite the presence of lack-of-fusion defects in samples produced at 67.2 W and 91.2 W, the post-heat treatments were performed to evaluate precipitation behavior, microstructural evolution, and mechanical response, thereby isolating the effects of thermal processing from densification-related defects.

To improve structural uniformity and enhance the overall properties of the samples, the as-built (green) specimens were annealed in a protective argon atmosphere using a furnace (Across International, model GCF-1400, Livingston, NJ, USA). Samples were heated from room temperature to the target temperature at a constant rate of 5 °C/min, held for 4 h, and then allowed to cool naturally within the furnace.

The post-heat treatment temperatures were selected by considering dilatometry measurements, the Cu-Ni-Si phase diagram, and previous studies on precipitation-hardenable copper alloys reported in the literature. Dilatometry was performed using a horizontal dilatometer, DIL L75 HX 1600 (LINSEIS, Selb, Germany).

The heat treatment parameters are summarized in Table 3.

### 2.4. Characterization

Material density was measured using the Archimedes method with an AD-1653 density determination device (HR Series, Seoul, Republic of Korea). To evaluate porosity and microstructural features, rectangular samples were cross-sectioned, embedded in epoxy resin, and polished perpendicular to the scanning plane. Polishing was performed using a universal grinding and polishing device (Qpol Go, ATM Qness GmbH, Mammelzen, Germany) with sandpapers of progressively finer grit sizes, ranging from 80 µm to 4 µm.

The microstructure of the raw material, LPBF-fabricated, and sintered samples was examined using a Prisma E Scanning Electron Microscope (SEM, Thermo Fisher Scientific, Hillsboro, OR, USA) equipped with an energy-dispersive spectroscopy (EDS) detector.

Electrical resistance was measured using the four-probe method, a technique that ensures precise measurements by eliminating errors associated with contact resistance, wire resistance, and other circuit-related factors. To minimize additional deviations, such as those arising from temperature effects, the direction of the applied current (*I*) was alternated. The difference in the measured voltage (Δ*V*) was then used to calculate the resistance using the sheet resistance.

(1) $$R_{S}=\frac{\pi }{ln2}\times \frac{\Delta V}{I}.$$

Correspondingly, the conductivity *σ* of the samples was calculated by

(2) $${\sigma =1/(d\times R}_{S}),$$

where *d* is the thickness of the sample.

The measurements were conducted using Cryomech model No. ST405, Syracuse, NY, USA (where four probe measurement technology is included). The voltage measurements were performed on the Keithley 2182A nanovoltmeter, Cleveland, OH, USA. A four-probe configuration was used to eliminate the contribution of contact resistance. Two sets of samples were prepared: annealed and green, with thicknesses of *d*_1_ = 0.60 mm and *d*_2_ = 0.52 mm, respectively. The distance between the probes in the measurement setup is *S* = 2.4 mm, and the thicknesses of the samples vary in the range of 0.60 mm–0.52 mm, which are much smaller than 40% of probe distances; thus, eliminating the need for any correction to the calculated resistance values.

The thermal conductivity was measured using the Transient Plane Source (TPS) method (TPS 2500 S, Hot Disk AB, Gothenburg, Sweden) with a double-spiral, Kapton-insulated sensor. The sensor was positioned between the polished surfaces of two sample pieces during measurement, and a heating power of 0.5 W was applied for 1 s.

The Vickers hardness of both green and heat-treated samples was measured using the FALCON 600G2FA, INNOVATEST Europe BV, Maastricht, The Netherlands. The indenter has a square base with a 136° angle between opposite faces, and a load of 1.0 kgf was applied to the indenter, with a dwell time of 10 s. The samples were measured at room temperature. Several microhardness measurements were taken in different positions to calculate the mean value.

## 3. Results and Discussion

### 3.1. Feedstock Characterization

**Figure 1:**
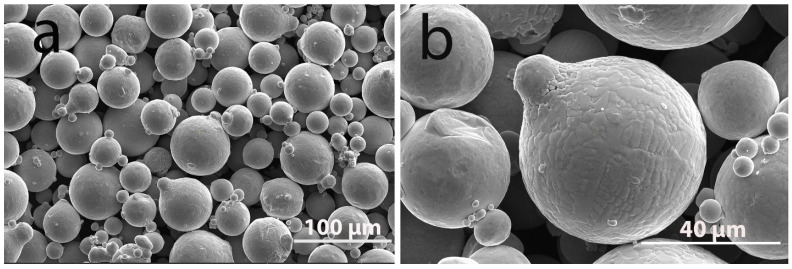
The SEM micrographs of the CuNi2.5SiCr powder at low (**a**) and high (**b**) magnifications.

The morphology of the CuNi2.5SiCr powder, as characterized by scanning electron microscopy and depicted in Figure 1, consists predominantly of spherical particles. This shape provides superior flowability and packing density to the CuNi2.5SiCr alloy powder. The attendant low surface area-to-volume ratio facilitates efficient melting and fusion during laser powder bed fusion, thereby improving densification, minimizing porosity, and enhancing the structural integrity of the resulting components [44]. Some particles featured adherent satellite particles on their surfaces, characteristics commonly observed in gas-atomized powders. The distribution of the powder on the build platform was examined visually prior to fabrication; necessary adjustments were made to the tension rubbers to ensure proper leveling.

The LPBF process was conducted employing a range of laser power levels. The densities of the resultant samples are summarized in Table 4.

All the other key processing parameters (exposure time of 10 µs, point distance of 10 μm, hatch spacing of 80 μm, and layer thickness of 40 μm) were held constant across the experiments. Specifically, the laser power ranged from 67.2 W to 100.8 W, yielding measured sample densities that increased from 8.432 to 8.600 g/cm^3^-equivalent to 95–98% of the theoretical density. The theoretical density of the CuNi2.5SiCr alloy (8.79 g/cm^3^) was used as the reference value, which was calculated using the rule of mixtures incorporating the weight percentages of its constituent elements and their individual densities. The relatively lower densities observed, particularly at suboptimal laser power settings, are a known challenge in additive manufacturing of copper alloys, often attributed to their high infrared reflectivity and thermal conductivity which can destabilize the melt pool [45]. This challenge often results in increased porosity if process parameters are not meticulously optimized, highlighting the critical role of precise control over laser-material interactions [24,46]. To address these challenges, a detailed examination of the microstructure in samples affected by laser power is essential.

### 3.2. Effect of Laser Power on Microstructure

**Figure 2:**
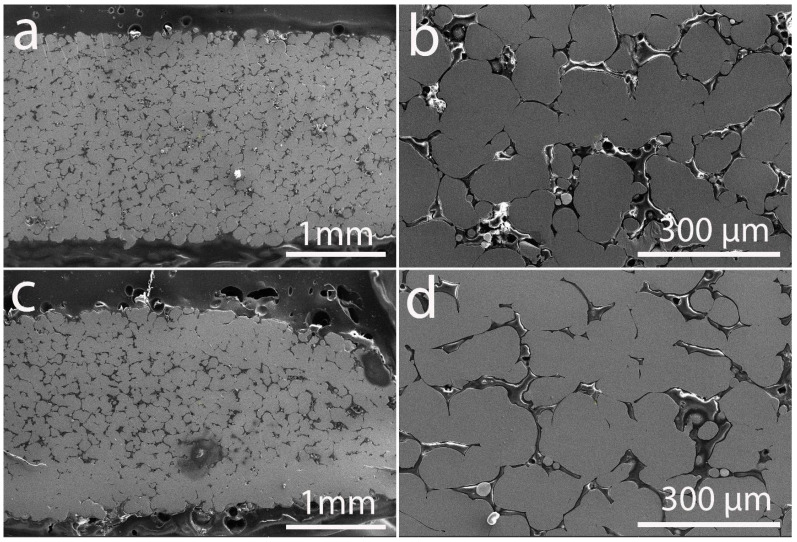
SEM micrographs of LPBF-fabricated (green) CuNi2.5SiCr samples produced at different laser powers: 67.2 W (**a**,**b**) and 91.2 W (**c**,**d**), shown at low (**a**,**c**) and high (**b**,**d**) magnifications.

Figure 2 illustrates the microstructure of LPBF fabricated CuNi2.5SiCr samples processed at two different laser powers: 67.2 W (Figure 2a,b) and 91.2 W (Figure 2c,d).

**Figure A1:**
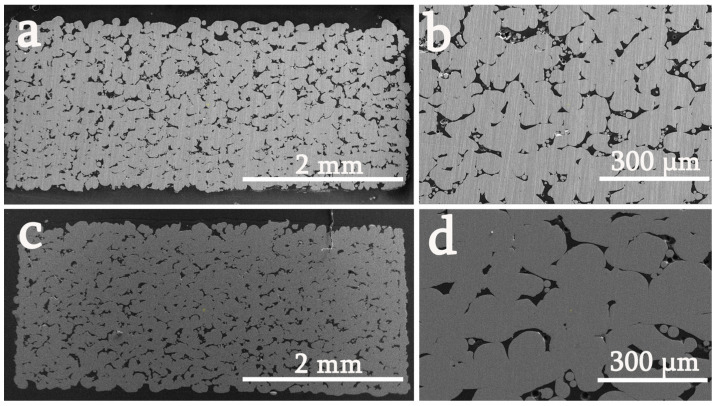
SEM micrographs of LPBF-fabricated (green) CuNi2.5SiCr samples produced at different laser powers: 96 W (**a**,**b**) and 100.8 W (**c**,**d**), shown at low (**a**,**c**) and high (**b**,**d**) magnifications.

Figure 2 shows the cross-sectional micrographs of the LPBF-fabricated CuNi2.5SiCr alloys processed at laser powers of 67.2 W and 91.2 W, while Figure A1 presents those at 96 W and 100.8 W (see Appendix A). These micrographs reveal distinct microstructural features and variations in grain morphology that are directly attributable to the differing energy inputs during the laser powder bed fusion process.

At a laser power of 67.2 W, the specimens display a uniformly porous microstructure. Although the sintering necks have formed between neighboring powder particles, complete densification has not been attained, leaving voids within the partially fused regions. The grains are relatively fine and equiaxed. Individual grains remain clearly distinguishable, and the inter-particle necks are comparatively small relative to the grain diameter. The grain size distribution is therefore narrow, with most grains exhibiting similar projected areas. Isolated grains measuring 5–40 μm are also present within pores.

This morphology indicates that the thermal input was insufficient to promote extensive grain boundary migration and grain coalescence. Under these conditions, consolidation proceeds mainly through localized melting and partial remelting at particle contacts, followed by rapid solidification. This indicates that the energy input was insufficient to enable complete melting and consolidation of the powder particles. This observation aligns with similar findings in LPBF of copper alloys, where inadequate energy density frequently results in incomplete fusion and significant residual porosity, despite localized sintering effects [47].

In contrast, at the higher laser power of 91.2 W, a substantial increase in grain size is evident (Figure 2c,d). The grains are larger, irregularly shaped with local elongations, and some original particles or grain boundaries are no longer distinctly discernible. This reflects extensive grain coalescence and boundary elimination, resulting in a broader and right-skewed grain size distribution. The presence of merged grains and larger contiguous solid domains suggests that the higher thermal energy and longer local melt-pool lifetimes enhanced grain boundary mobility and promoted curvature-driven grain growth.

However, at a laser power of 91.2 W, the microstructure exhibits non-uniformity, with the region adjacent to the substrate appearing highly dense while the upper layers remain insufficiently fused. This inhomogeneity can be attributed to the formation of a molten copper alloy possessing elevated viscosity. The gravity drove the melt to flow down, which led to depression in the center of the melt pool after solidification. This caused formation of defects and voids, which created more defective layers. A similar phenomenon has been reported by Mo et al. [48].

Under the low laser power condition, pores are mostly small, rounded, and uniformly distributed throughout the microstructure (Figure 2b). Most occur at grain junctions and between grains, forming an interconnected but constrained pore network. These pores arise from lack-of-fusion defects due to insufficient energy density. Limited melt-pool size and short lifetime prevent pore migration and coalescence, maintaining a fine, homogeneous pore population.

In contrast, the microstructure processed at higher laser power exhibits a markedly broader pore size distribution, encompassing large and irregular pores that are frequently elongated along grain boundaries and locally coalesced into cavities (Figure 2a). The expanded and more dynamic melt pool enhances pore mobility and coalescence, thereby promoting pore growth and coarsening. Although majority pores are still indicative of lack-of-fusion defects, the presence of enlarged cavities indicates that unstable melt-pool dynamics and partial keyholing may contribute additionally under these conditions.

Increasing the laser power to 96 W and 100.8 W (Figure A1) reveals a clear transition to a coarser and more heterogeneous microstructure. Higher powers promote pronounced grain coalescence and partial boundary elimination, yielding larger, locally elongated grains and a broader, right-skewed grain size distribution. This grain coarsening reflects the increased melt pool size and lifetime at higher energy input, which, despite the intrinsically high thermal conductivity of the CuNi2.5SiCr alloy, enables enhanced grain boundary mobility and post-solidification coarsening. Concurrently, pore morphology shifts dramatically: low laser power conditions feature fine lack-of-fusion pores at grain junctions, whereas high-power samples contain larger, irregular pores. As a result, grain growth and pore coarsening are strongly coupled in the higher laser power condition, leading to a less uniform microstructure with locally clustered large pores, whereas the lower laser power condition retains a finer, more homogeneous grain structure and a narrower pore size distribution.

Given the observed microstructural evolution characterized by finer grains and more uniform porosity at lower laser powers and the absence of significant density variations in LPBF-fabricated samples up to 100.8 W, post-processing is recommended for samples produced at 67.2 W to mitigate coarsening and pore enlargement from excessive thermal input.

### 3.3. Heat Treatment of LPBF-Fabricated Samples

**Figure A2:**
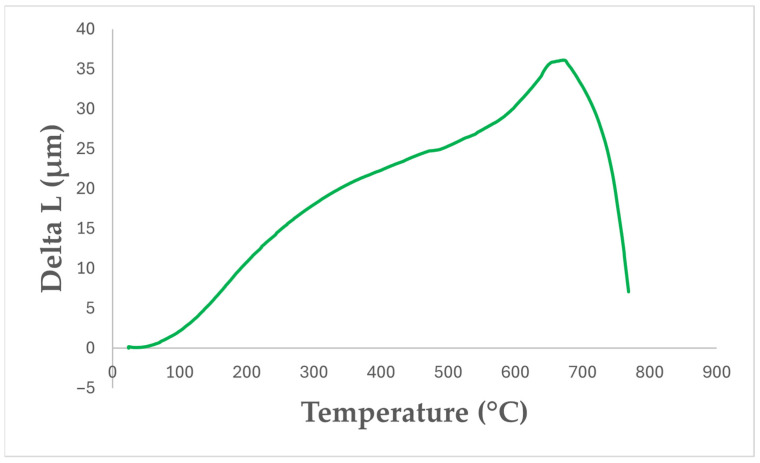
Temperature-dependent dilatometry curve of the CuNi2.5SiCr alloy.

The LPBF-fabricated samples produced at a laser power of 67.2 W underwent subsequent heat treatment. Dilatometry was conducted to identify the optimal temperature, with the results presented in Figure A2 (see Appendix A). A nearly linear and weak dimensional expansion is observed up to 650 °C. Above this temperature, the dilatation reverses and a rapid contraction is observed between about 660 and 740 °C. Based on these findings, the samples were heated linearly at 5 °C/min to 750 °C and held for 4 h. Following the analysis of the results obtained, the heat treatment temperature was increased to 850 °C and 950 °C.

**Figure 3:**
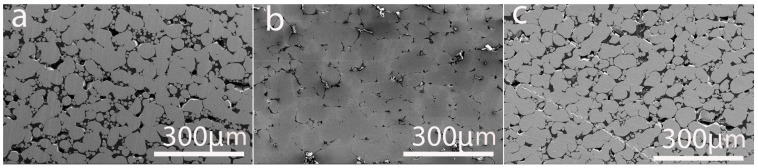
Microstructure of LPBF-fabricated samples using 67.2 W laser power after the post-heat treatment for 4 h at (**a**) 750 °C, (**b**) 850 °C, and (**c**) 950 °C.

Figure 3 illustrates the microstructural evolution of the samples under different heat treatment conditions. The microstructures of the heat-treated copper samples exhibit clear and systematic evolution with increasing annealing temperature, reflecting the progressive dominance of recovery, recrystallization, and grain growth mechanisms.

At 750 °C (Figure 3a), The grains appear irregular in shape and smaller compared to samples before heat treatment. This indicates that recrystallization has initiated but is not yet fully complete. Numerous grain boundary junctions are visible, and subtle contrast variations within individual grains suggest residual substructures such as low-angle boundaries and dislocation networks. This microstructure is characteristic of an early to intermediate recrystallization stage, where new strain-free grains have nucleated but their growth is constrained by limited atomic mobility at this temperature. Moreover, distinct precipitates at grain boundaries and within grain interiors-particularly phases like Ni-Si and Cr-Si phases act as pinning points for grain boundaries, impeding growth and refining the overall structure, which ultimately affects the properties of the Cu-Ni-Si-Cr alloy [49,50]. As reported in the literature, the simultaneous presence of nickel silicide phases impedes dislocation motion and enhances strength in the Cu-Ni-Si-Cr system [51].

At 850 °C (Figure 3b), the microstructure becomes noticeably coarser and more homogeneous. Grain boundaries are smoother and more clearly defined, and the fraction of small grains is significantly reduced. The grains show a more uniform equiaxed morphology, indicating that recrystallization is essentially complete, and that grain growth has become the dominant process. Compared to 750 °C, the average grain size is substantially larger, reflecting enhanced boundary mobility and increased diffusion rates at this higher temperature. Furthermore, the increased thermal energy at 850 °C facilitates the dissolution of smaller precipitates and the coarsening of larger ones, which can further impact grain growth kinetics. As a result, a denser microstructure with significantly reduced intergranular porosity is formed, indicating a more advanced sintering stage.

**Figure 4:**
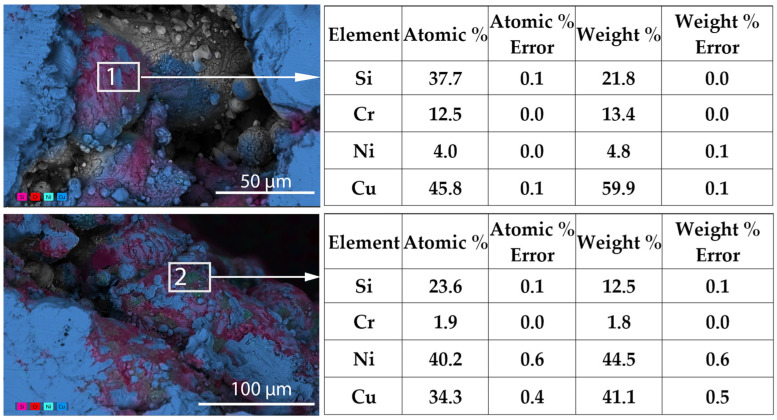
EDS analysis results of the LPBF-fabricated samples after the heat treatment.

The EDS analysis results for the heat-treated samples are provided in Figure 4, where the accumulation of nickel silicide in the matrix after heat treatment is observed.

This observation aligns with previous research indicating the formation of Ni-Si and Cr-Si precipitates in Cu-Ni-Si-Cr alloys, which are critical for densification and strengthening mechanisms [52,53]. These secondary phases are predominantly precipitated along grain boundaries, where they facilitate enhanced sintering through localized softening and diffusion [54]. Their presence supports improved grain bonding and contributes to the overall densification process observed at elevated heat treatment temperatures.

Elevating the annealing temperature to 950 °C induces substantial grain growth, but noticeable porosity remains, with smaller, persistent voids still present (Figure 3c). The increased energy at this temperature accelerates the dissolution rate of the nickel silicide phases within the copper matrix. This leads to coarsening of the alloy grains. Grain boundaries appear relatively smooth and widely spaced, and the overall boundary area per unit volume is significantly reduced. Such extensive grain growth is typical at very high annealing temperatures, where diffusion is rapid and grain boundary mobility is maximized.

### 3.4. Mechanical, Thermal and Electrical Properties

**Figure 5:**
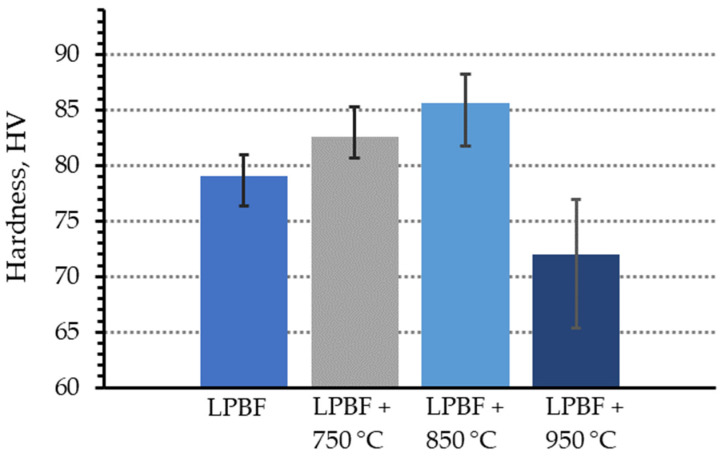
Variation in Vickers hardness of CuNi2.5SiCr alloy samples fabricated by LPBF and subjected to post-heat treatment at different temperatures.

The Vickers microhardness of both as-fabricated and post-heat-treated samples was evaluated. The LPBF sample produced at a laser power of 67.2 W exhibited an average hardness of 79 HV1.0 in the as-fabricated condition. After annealing at 850 °C, the hardness increased to 85 HV1.0 (Figure 5).

However, a further increase in the post-treatment temperature resulted in a progressive reduction in hardness, reaching 72 HV1.0 after annealing at 950 °C. The highest hardness values were obtained for the specimens exhibiting the highest relative density, whereas the presence of porosity led to a noticeable deterioration in the measured mechanical response.

CuNi2.5SiCr is a precipitation-hardenable alloy, and its hardness is governed not only by porosity and dislocation density introduced during LPBF processing, but also by the formation and evolution of fine strengthening precipitates. Annealing at 850 °C promotes the precipitation of fine Cu-Ni-Si phases, which effectively hinder dislocation motion and thereby enhance the hardness. This precipitation-strengthening effect coincides with the attainment of the maximum relative density, explaining the observed peak in microhardness at this temperature.

At higher annealing temperatures, the precipitates undergo coarsening and partial dissolution, while recovery and stress relaxation become more pronounced. These microstructural changes reduce the effectiveness of precipitation strengthening and decrease the overall resistance to plastic deformation, which accounts for the drop in hardness observed at 950 °C.

Overall, the present results are consistent with previously reported behavior of precipitation-strengthened copper alloys processed by laser powder bed fusion, in which an optimum post-heat-treatment temperature leads to peak hardness, followed by a decline associated with precipitate coarsening and over-aging [35,55,56,57,58].

The literature indicates that, even with a high laser power of 370 W, the Vickers microhardness of LPBF-processed CuNi2.5SiCr alloy is approximately 85 HV (under a 500 g load) [59]. Intense subsequent heat treatment, which promotes recrystallization and formation of ultrafine grains, yields substantially higher hardness values of up to ~115 HV. This disparity emphasizes the critical role of understanding the complex interplay between the processing parameters, microstructure, and mechanical behavior in additively manufactured copper alloys. Further research into the underlying mechanisms of precipitate evolution and grain growth kinetics across diverse thermal conditions is essential to optimize the mechanical performance of CuNi2.5SiCr alloys for additive manufacturing applications.

Thermal conductivity measurements were carried out on the samples heat-treated at 850 °C, which exhibited a value of 20 W/m·K.

**Figure 6:**
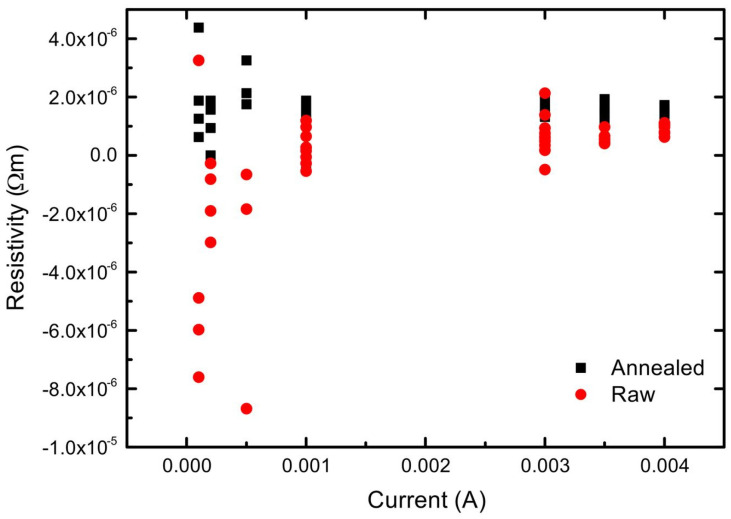
Deviation of conductivity depending on the current applied on green and annealed samples.

Electrical conductivity was determined using DC current-voltage measurements. To avoid heating effects the measurements were started at low currents. Because the samples exhibit relatively low resistance, measurements performed at very low currents (≤0.5 mA) produced voltage signals close to the instrument resolution limit. Under these conditions, the relative influence of electrical noise, thermal fluctuations, and instrumental resolution limits dominated the measurement uncertainty at very low currents and led to large scatter in the calculated conductivity values (Figure 6).

To improve the signal-to-noise ratio, the applied current was gradually increased. As the current increased from 0.1 mA to 1 mA, the measurement deviation decreased, indicating that the voltage signal became less affected by background noise and parasitic effects. Additional measurements were therefore conducted at higher current levels (3–4 mA). In this range, the calculated conductivity values showed minimal variation with current, confirming that the measurements were performed in the linear ohmic regime and that self-heating effects were negligible. A current of 4 mA was selected for the final measurements, as it provided the most stable and reproducible results.

To eliminate remaining influence of thermoelectric effect, voltage measurements were performed with current applied in both forward and reverse directions. For each sample, 20 voltage readings were recorded (10 in each polarity), and the resistance was determined from the averaged voltage difference (Formula (1)). The electrical conductivity was then calculated based on the sample geometry. The mean conductivity values and their standard deviations are summarized in Table 5.

The green LPBF-ed sample exhibits a higher electrical conductivity (*σ* = 1.07 × 10^6^ S/m) compared to the annealed one (LPBF + 850 °C), which shows a conductivity of *σ* = 0.66 × 10^6^ S/m. The lower conductivity of the annealed material is attributed to the formation of silicide-based precipitates at grain boundaries, which increase electron scattering. Nevertheless, both materials have conductivities far below that of pure copper (5.8 × 10^7^ S/m), which remains superior for applications where minimal resistive losses are required.

Although annealing typically enhances conductivity in Cu alloys by eliminating dislocations and minimizing defect scattering, CuNi2.5SiCr samples in this study displayed the opposite effect. In many conventionally processed Cu alloys, conductivity increases after heat treatment due to precipitation processes that reduce lattice defects and improve electron transport pathways [60,61]. However, those studies differ fundamentally from the present work in both processing route and heat-treatment conditions.

The alloys reported in the literature were usually produced by conventional methods and annealed at moderate temperatures (450–500 °C) for long durations (12–25 h). In contrast, the CuNi2.5SiCr samples here were fabricated by low-power laser powder bed fusion, which results in rapid solidification, melt-pool boundaries, micro-segregation, and residual porosity-microstructural features that are less pronounced in conventionally processed materials. Furthermore, annealing in this work was performed at a higher temperature but for a shorter duration (4 h), leading to different phase evolution kinetics.

These differences can strongly influence the observed outcomes. Additive manufacturing microstructures often exhibit melt-pool boundaries, rapid solidification micro-segregation, and residual porosity-features especially prevalent in low-power processes. Such defects generally reduce conductivity compared to conventionally produced alloys, e.g., Wang et al. reports 11–21 MS/m conductivity for similar alloys [60,61,62]. While annealing can increase conductivity in many cases, sometimes it may also lead to opposite tendency if:

1. There is oxide formation at grain boundaries promoted by residual oxygen trapped in pores and from small amount of oxygen contained in argon atmosphere, which introduces electrically resistive interfaces, or
2. Inter-metallic phases (Ni-Si) develop at boundaries.

In current study the EDS analysis (Figure 4) confirms segregation of Ni and Si in these regions after heat treatment. Such inter-metallic phases increase electron scattering and can exhibit semiconducting or poorly conductive behavior, thereby reducing overall electrical conductivity.

As a result, the heat treatment in the present additively manufactured CuNi2.5SiCr alloy promotes boundary-phase formation rather than defect recovery alone, leading to a net decrease in electrical conductivity.

## 4. Conclusions

CuNi2.5SiCr alloy was successfully fabricated by LPBF using a low laser power range of 67.2–100.8 W. Relative densities of 95.9–97.8% were achieved, demonstrating that high densification is possible for this alloy even at low laser power when suitable processing parameters are applied. Laser power strongly affected microstructure and defect morphology: low power (67.2 W) produced finer, more homogeneous grains with small and uniformly distributed lack-of-fusion pores, whereas higher powers promoted grain coarsening, pore growth and local microstructural inhomogeneity due to unstable melt-pool behavior.

The post-heat treatment of samples fabricated at 67.2 W resulted in systematic microstructural evolution. Annealing at 850 °C yielded a more homogeneous and denser microstructure and promoted the formation of Ni-Si- and Cr-Si-based precipitates, while treatment at 950 °C led to excessive grain growth and persistent residual porosity. The maximum hardness of 85 HV1.0 was obtained after annealing at 850 °C, compared with 79 HV1.0 in the as-fabricated condition, whereas over-annealing at 950 °C reduced the hardness to 72 HV1.0 due to precipitate coarsening and recovery. The thermal conductivity of the optimally heat-treated material was 20 W/m·K.

Overall, the results show that a low-power LPBF strategy combined with an appropriate post-heat treatment enables effective densification and precipitation strengthening of CuNi2.5SiCr, providing mechanical performance comparable to that reported for significantly higher laser powers.

Stable and reproducible electrical conductivity measurements were achieved at an applied current of 4 mA, which ensured operation in the linear ohmic regime and minimized noise and thermoelectric effects. The as-fabricated LPBF sample exhibited a higher electrical conductivity (1.07 × 10^6^ S/m) than the sample annealed at 850 °C (0.66 × 10^6^ S/m).

The decrease in conductivity after the heat treatment is mainly attributed to the formation and segregation of Ni-Si-based precipitates at grain boundaries, which increase electron scattering, as confirmed by EDS analysis. In contrast to conventionally processed Cu-Ni-Si alloys, the LPBF microstructure and the high-temperature, short-duration annealing promote boundary-phase formation rather than defect recovery, leading to a net reduction in electrical conductivity.

## Figures and Tables

**Table 1:** Summary of studies on processing copper and alloys using LPBF.

Composition of Feedstock	Relative Density Achieved	Laser Power	Layer Thickness	Hatch Spacing	Ref.
Gas-atomized pure copper powder	88.1%	200 W	50 μm	120 μm	[25]
Pure copper powder: 99.9% Cu, 0.010% P, and 0.005% O	99.6%	800 W	-	25–120 μm	[26]
Gas-atomized pure copper powder	99.10%	300 W	30 μm	80 μm	[27]
Gas-atomized high-purity 99.9% copper powder	96.6%	800–900 W	50 μm	100 μm	[28]
Gas-atomized copper powder with 99.95%	98%	600–800 W	30 μm	70 μm and 90 μm	[17]
Gas-atomized commercial Cu powders	99%	300 W	30 μm	80 μm	[29]
Gas-atomized Cu-Cr-Zr copper alloy	99.14%	370 W	30 μm	120 μm	[30]
Cu-Cr-Zr alloy powder	99.43%	425 W	30 μm	90 μm	[31]
Cu-Cr-Zr alloy powder	99.99%	450 W	40 μm	50 μm	[32]
Cu-Ni_2_-Si-Cr alloy powder	99.5%	270 W	30 µm	110 μm	[33]
Cu-Cr1-Zr alloy powder	99.84%	370 W	20 µm	100 μm	[34]
Cu-Cr-Zr alloy powder	>99%	400 W	30 μm	70 μm	[35]

**Table 2:** Nominal chemical composition of CuNi2.5SiCr powder.

Element	Ni	Si	Cr	Other Elements	Cu
wt. %	2․5	0․65	0.3	max 0.3	balance

**Table 3:** Heat treatment conditions for the green samples.

Scheme Number	Heat Treatment Temperature, °C	Dwell Time, h
1	750	4
2	850	4
3	950	4

**Table 4:** Archimedes density and relative density values of CuNi2.5SiCr samples fabricated by LPBF at different laser powers.

Laser Power, W	Measured Density by Archimedes, g/cm^3^	Relative Density, %
67.2	8.504	96.8
72	8.432	95.9
79.2	8.598	97.8
84	8.455	96.2
91.2	8.600	97.8
96	8.564	97.4
100.8	8.555	97.3

**Table 5:** Electrical properties of annealed and green samples of CuNi2.5SiCr.

Data	N Total	Mean/S ÷ m	Standard Deviation
Conductivity (LPBF + 850 °C)	10	0.66219 × 10^6^	4.5754 × 10^4^
Conductivity (LPBF)	10	1.06995 × 10^6^	2.3615 × 10^5^

## Data Availability

The original contributions presented in this study are included in the article. Further inquiries can be directed to the corresponding author.
